# The structural equation model on self-efficacy during post-op rehabilitation among non-small cell lung cancer patients

**DOI:** 10.1371/journal.pone.0204213

**Published:** 2018-09-20

**Authors:** Fei Fei Huang, Qing Yang, Juan Zhang, Xuan Ye Han, Jing Ping Zhang

**Affiliations:** 1 School of Nursing, Fu Jian Medical University, Fu Zhou, China; 2 Department of Anesthesia, Massachusetts General Hospital, Boston, Massachusetts, United States of America; 3 Department of Cardiothoracic Surgery, Fuzhou General Hospital of Nanjing Military Command, Fu Zhou, China; 4 Department of Neurosurgery, Second Affiliated Hospital of Harbin Medical University, Harbin, China; 5 Psychological Nursing Research Center, Xiangya School of Nursing, Central South University, Changsha, China; ITALY

## Abstract

**Backgrounds:**

Self-efficacy plays an important role in pulmonary rehabilitation, but it is still unknown which factors exert their effects on postsurgical rehabilitation self-efficacy among non-small cell lung cancer (NSCLC) patients. This study aims to assess relationships among physical function, social factors, psychological factors, quality of life (QOL) and self-efficacy, and the effects of these variables on self-efficacy among NSCLC patients.

**Methods:**

A cross-sectional survey was administered to 238 postsurgical NSCLC patients (response rate 95.2%) at five tertiary hospitals in Fuzhou, China. the participants completed a pack of questionnaires. Structural equation modeling (SEM) was conducted to test the hypothetical model.

**Results:**

The SEM results supported the hypothesized structural model (χ2/df = 1.511, *p>*0.05). The final model showed that confrontation coping, subjective well-being (SWB), social support, psychological growth (PTG) and anxiety and depression can be directly related to self-efficacy (coefficient = 0.335, coefficient = 0.288, coefficient = 0.150, coefficient = 0.024, and coefficient = -0.004, respectively, *p*<0.01). Confrontation coping also had indirect effect via SWB (coefficient = 0.085, *p*<0.01), which had indirect connection via PTG (coefficient = 0.005, *p*<0.01). Social support and anxiety and depression had indirect pathways as well. As expected, self-efficacy directly affected the quality of life.

**Conclusions:**

This study suggests that higher confrontation coping style, SWB, social support, and PTG and lower anxiety and depression levels could effectively enhance their self-efficacy and consequently, improve QOL. These findings may help develop an intervention aimed at enhancing self-efficacy for this patient population.

## Introduction

Lung cancer is a leading cause of cancer mortality and morbidity worldwide [1]. In China, the incidence of lung cancer has markedly increased over the last few decades and is predicted to be 1 million per year by 2025 [2,3]. Presently, surgery remains the primary treatment for lung cancer [4]. Pulmonary rehabilitation management was found to effectively reduce the symptom burden and side effects after pneumonectomy, such as pain [5], fatigue [6], anxiety and depression [7], and increase lung function [8,9] and the quality of life (QOL) [10,11].

Self-efficacy plays an important role in pulmonary rehabilitation [12]. Signe et al. demonstrated that self-efficacy was one of the predictors of health status improvement and overall QOL in pulmonary rehabilitation [13]. Previous studies have investigated the relationship between self-efficacy and numerous cancer-related factors, including socio-demographic and clinical characteristics, physical function, psychological distress (e.g., anxiety and depression), psychological growth (i.e. post-traumatic growth, PTG), health behaviors and QOL [4,14–16]. However, the pathway or mechanism associating self-efficacy with physical or psychological health outcomes on cancer patients is not well understood. Furthermore, these previous studies have demonstrated such relationships through the use of self-efficacy as a mediator or independent variable, not as a dependent variable. A structural equation modeling (SEM), which analyzes both direct interrelationships of independent variables and their indirect effects through other variables [17], may help clarify the role of self-efficacy in lung cancer patients.

According to the definition set forth by Bandura, self-efficacy is a belief in one’s ability to organize and execute the course of action required to produce a specific outcome, usually specific to a given task or domain [18]. Self-efficacy for coping and management may change depending on the type of cancer and phases of care from diagnosis, to treatment and survivorship [19]. So far, majority of the studies looked at breast and colorectal cancer and few focused on lung cancer, especially non-small cell (NSCLC) to identify the relationships and interactions between self-efficacy and physical function, social factors, psychological factors and QOL among NSCLC patients during post-op rehabilitation.

Given the high disease burden of lung cancer in China, and the importance of self-efficacy in pulmonary rehabilitation, it is imperative to identify pathways through which factors exert their effects on postsurgical rehabilitation self-efficacy, which can then be applied to design integrated interventions to enhance self-efficacy and QOL. The aim of this study was to use SEM to determine factors that may affect self-efficacy in postsurgical NSCLC patients. The following hypotheses are proposed and tested to generate a behavioral relationship model:

1. Pain, poor sleep condition, unproductive coping strategies (fear-avoidance and acceptance-resignation), and anxiety and depression will have a direct negative relationship to self-efficacy.
2. Confrontation coping, social support, SWB, and PTG will be have a direct positively relationship to self-efficacy.
3. Self-efficacy will have a direct positive relationship with QOL.

## Methods

### Study design

From March 2015 to August 2015, a cross-sectional study was conducted in the departments of cardiothoracic surgery at five tertiary hospitals in Fuzhou, Fujian province, China. Two hundred fifty patients who satisfied the following inclusion criteria were enrolled: a) able to read and understand Chinese, b) age ≥18, c) have undergone lung resection for suspected or confirmed lung cancer, d) Karnofsky performance status score ≥60%. Participants with cognitive deficits, severe postoperative complications, metastasis or other malignancies were excluded. A sample size greater than 200 was required for SEM analysis [20,21]. Clinical data were collected from the medical records and all participants filled out a set of structured questionnaires (see below). Before survey, all eligible participants included in this study were given verbal informed consent. This study was approved by the Institutional Review Board of Central South University (number: 2013017). (S1 Fig and S1 File)

### Measurements

#### Demographic and clinical characteristics

The demographic data collected included age, gender, marital status, place of residence, educational level, religion, family per capita monthly income (Yuan, RMB), employment status, medical insurance, smoking status. Clinical information collected from the chart review included surgical site, cancer stage, histological type, type of surgery, length of hospital stay and comorbidities.

#### Postoperative rehabilitation self-efficacy

The self-efficacy of postoperative rehabilitation management was measured using the Self-Efficacy Scale for Postoperative Rehabilitation Management of Lung Cancer (SESPRM-LC) [4]. The 27-item SESPRM-LC with six sub-domains was rated on a five-point Likert scale (1 = *not at all confident* to 5 = *completely confident*). The total scores of SESPRM-LC ranged from 27 to 135, with higher scores indicating greater self-efficacy. The reliability and validity have demonstrated sound psychometrics in NSCLC postsurgical patients through the classical test theory and item response theory methods [4].

#### Quality of life (QOL)

QOL was assessed using the Functional Assessment of Cancer Therapy–Lung scale (FACT-L, Version 4) [22], which has been widely used in lung cancer studies with adequate psychometric properties. FACT-L contains 36 items covering the factors of well-being in the physical, functional, emotional, social/family realms, and lung cancer subscale. All FACT-L items are rated on a 5-point Likert-type scale ranging from 0 (not at all) to 4 (very much). Higher scores represent better quality of life or fewer symptoms[22]. In this study, the Cronbach’s α coefficients for the five subscales ranged from 0.543 to 0.932, and for the overall scale 0.896.

#### Anxiety and depression

Anxiety and depression were measured by the Chinese version of the Hospital Anxiety and Depression scale (HADS)[23]. The HADS contains 14 items for anxiety and depression. Each subscale is scored from 0 to 21, with higher scores indicating greater distress. This score is classified as no anxiety or depression (0–7), borderline (8–10), and severely anxious/depressed (11–21). The Chinese version of HADS has been validated [23]. In this study, the Cronbach’s α coefficients for the five subscales ranged from 0.686to 0.750, and for the overall scale 0.817.

#### Social support

Social support was measured by the Multidimensional Scale of Perceived Social Support(MSPSS)[24,25]. The 12-item MSPSS contains 3 factors, the perceived social support from family, friends, and significant others. The scale employed a 7-point rating scale ranging from 1 (Very strongly disagree) to 7 (Very strongly agree). In this study, the Cronbach's α coefficient was 0.907 for overall scale.

#### Subjective well-being (SWB)

SWB was assessed by the Simplified Face Scale. This very brief, pictorial scale of mood uses a sequence of seven faces and does not require reading literacy [26,27]. The seven faces range from happy and smiling to sad and crying. In this study, the Cronbach's α coefficient was 0.891 for overall scale.

#### Coping

Coping styles were measured with the Medical Coping Modes Questionnaire (MCMQ) [28,29]. A 19-item MCMQ was used to evaluate patients’ cognitive-behavioral and illness-related coping strategies, including confrontation, fear-avoidance and acceptance-resignation. The scale is scored on a four-point Likert scale ranging from 1 (never) to 4 (very much). In this study, the Cronbach's α coefficient of overall scale was 0.762.

#### Sleep condition

Sleep condition was measured by Athens Insomnia Scale(AIS) [30]. The AIS is a self-assessment psychometric instrument designed for quantifying sleep difficulty. It consists of 8 items measured on numeric scale (0 “no problem” to 3 “did not sleep at all”). The total score is classified as no sleep disorder (<4), suspected sleep disorder (4–6), and insomnia (>6). In this study, the Cronbach's α coefficient was 0.848.

#### Pain

Pain was measured by the visual analogue scale(VAS). which was considered the most accurate and reproducible scale of measuring the intensity of pain[31]. VAS was a numeric scale based on 11 points (0 “no pain” to 10 “the most intense and unbearable pain”). The psychometric properties were verified by prior studies [32].

#### PTG

PTG was measured by the Posttraumatic Growth Inventory (PTGI) [33]. The Chinese version of PTGI is a 21-item self-reported inventory with five domains, which are relation to others, new possibilities, personal strengths, spiritual change, and appreciation of life. The questionnaire uses a six-point scale (0 “I did not experience this change after the traumatic event” to 5 “I experienced this change to a very great degree after the traumatic event”). Higher scores implied greater posttraumatic growth. The Chinese version of PTGI was verified previously[34].In this study, the Cronbach’s α ranged from 0.701 to 0.850 for the five domains, and 0.926 for the overall score.

### Statistical analysis

Data analysis was conduct using SPSS 17.0 (IBM, Chicago, IL,USA) and Mplus 6.1. Missing data were managed by expectation maximization. *p*<0.05 was considered significant. Descriptive statistics were calculated to estimate the frequencies, means, and standard deviations of the study variables. Correlations among the factors were examined by Pearson’s correlation analysis and stepwise linear regression analysis. In this study, the data meet the assumptions of normality (one-sample kolmogrov-smirnov test was no statistical significance). SEM with a maximum likelihood (ML) estimation method was used to evaluate the fit of the hypothesized theoretical model based on the following criteria [35]: Normed chi-square (χ2/df, 1.0–3.0, *p*>0.05), Root Mean Squared Error of Approximation (RMSEA<0.08), Comparative Fit Index (CFI,>0.9), Normed Fit Index (NFI, >0.9), and Goodness of Fit Index (GFI, >0.9). Hypotheses regarding the structural relationships of the constructs in the final model were evaluated using the magnitude of path coefficients (standardized coefficient) and their significance. The bootstrap method was applied for the indirect effects in the hypothesized model (repeated 1000 times) using ML estimation.

## Results

### Characteristics of the participants

Of the 250 eligible patients recruited, 12 were excluded from analysis because of missing data > 30%. Four questionnaires contained missing data to a lesser degree and were included in the analysis. Final sample size was 238 patients (effective response rate 95.2%). Demographic and clinical characteristics of the participants are shown in Table 1. The participants’ age range was 32 to 68 years, and the average age was 58.37±9.94 years. Three-quarters were male. The length of hospital stay was 11.51±8.01 days. Vast majority (96%) of the participants were married.

**Table 1:** Demographic and clinical characteristics of study participants(N = 238).

Variable	N(%)	Variable	N(%)
Demographic data		Clinical characteristics	
Age (years)		Cancer stage (%)	
≤34	7(3.0%)	In situ	12(5.0%)
35~59	114(48.0%)	I (Ia+Ib)	86(36.1%)
>60	117(49.0%)	Ⅱ(Ⅱa+Ⅱb)	100(42.1%)
Gender (%)		Ⅲ(Ⅲa+Ⅲb)	40(16.8%)
Male	178(75.0%)	Histologic type(%)	
Female	60(25.0%)	Squamous	92(38.6%)
Place of residence(%)		Adenocarcinoma	131(55.0%)
Urban	94(39.5%)	Adenosquamous	9(3.8%)
Suburban	32(13.4%)	Bronchoalveolar	3(1.3%)
Rural	112(47.1%)	Large undifferentiated	3(1.3%)
Religion (%)		Type of surgery (%)	
Yes	117(49.2%)	Thoracotomy	86(36.1%)
No	121(50.8%)	VATS^a^	152(63.9%)
Educational level(%)		Comorbidities	
Less than high school degree	86(36.2%)	No	140(58.8%)
High school degree (including technical training)	100(42.0%)	Yes	98(41.2%)
Bachelor’s degree or higher	52(21.8%)	Surgical site	
Family per capita monthly income (Yuan, RMB)		Left	100(42.0%)
<1000	14(5.9%)	Right	134(56.3%)
1000~2999	90(37.8%)	Both	4(1.7%)
3000~4999	70(29.4%)	Re-admission	
5000~	64(26.9%)	Yes	56(23.5%)
Employment status		No	182(76.5%)
Full-time Employment	37(15.5%)		
Unemployment	11(4.6%)		
Retired	82(34.5%)		
Farmer	92(38.7%)		
Other	16(6.7%)		
Medical insurance(%)			
New agricultural cooperative medical insurance	135(56.7%)		
Urban basic medical insurance	98(41.2%)		
Self-paid(uninsured)	5(2.1%)		
Smoking status(%)			
Former/current smoker	115(48.3%)		
Never smoked	123(51.7%)		

a, VATS,Video-assisted thoracoscopic surgery.

### The hypothesis model of postsurgical rehabilitation management self-efficacy

**Fig 1:**
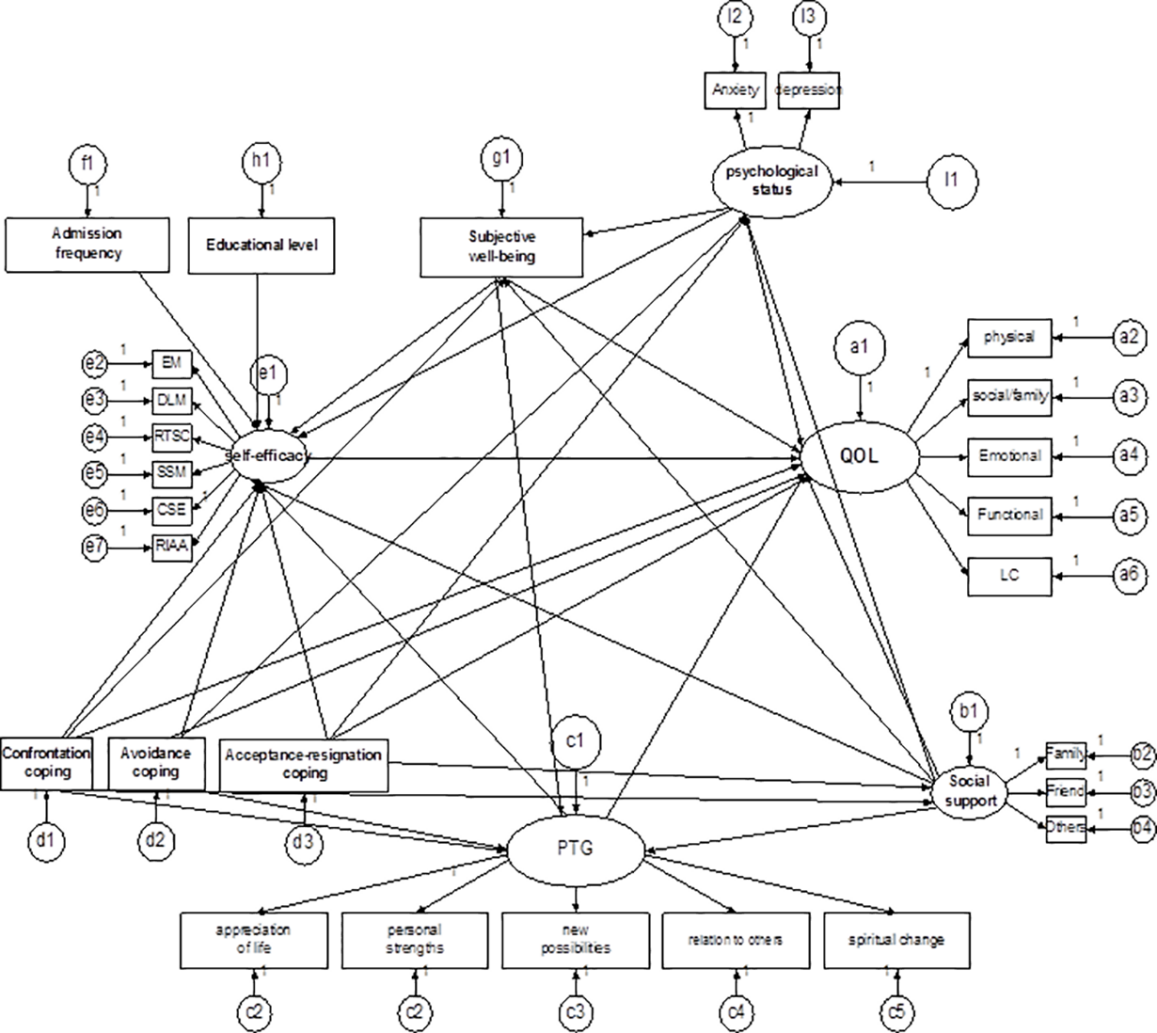
The hypothesis model of factors influencing self efficacy during postsurgical rehabilitation management. PTG, posttraumatic growth; LC, lung cancer subscale; EM, emotional management self-efficacy subscale; DLM, daily life management self-efficacy subscale; RTSC, rehabilitation training and skills cultivating self-efficacy subscale; RIAA, rehabilitation information acquisition and application self-efficacy subscale; CSES, coping with treatment side effects self-efficacy self-efficacy subscale; SSM, symptom self-management self-efficacy subscale. QOL, quality of life.

Based on the findings of previous study [36], 12 variables were included in the linear regression model. The results from stepwise linear regression analysis highlighted the six influencing factors: anxiety and depression (*Beta* = -0.247,adjusted *R*^*2*^ = 0.113, *p* = 0.004), social support (*Beta* = 0.768,adjusted *R*^*2*^ = 0.148, *p* = 0.000), SWB (*Beta* = 0.375, adjusted *R*^*2*^ = 0.136, *p* = 0.000), PTG (*Beta* = 0.300, adjusted *R*^*2*^ = 0.051, *p* = 0.029), confrontation and acceptance-resignation coping (*Beta* = 0.442 & -0.158, adjusted *R*^*2*^ = 0.202, *p*<0.01). Based on our understanding of the literature and these preliminary data, we constructed the hypothesis model for self efficacy during postsurgical pulmonary rehabilitation which was further analyzed with SEM (Fig 1).

### The final model and model estimation of SEM

**Fig 2:**
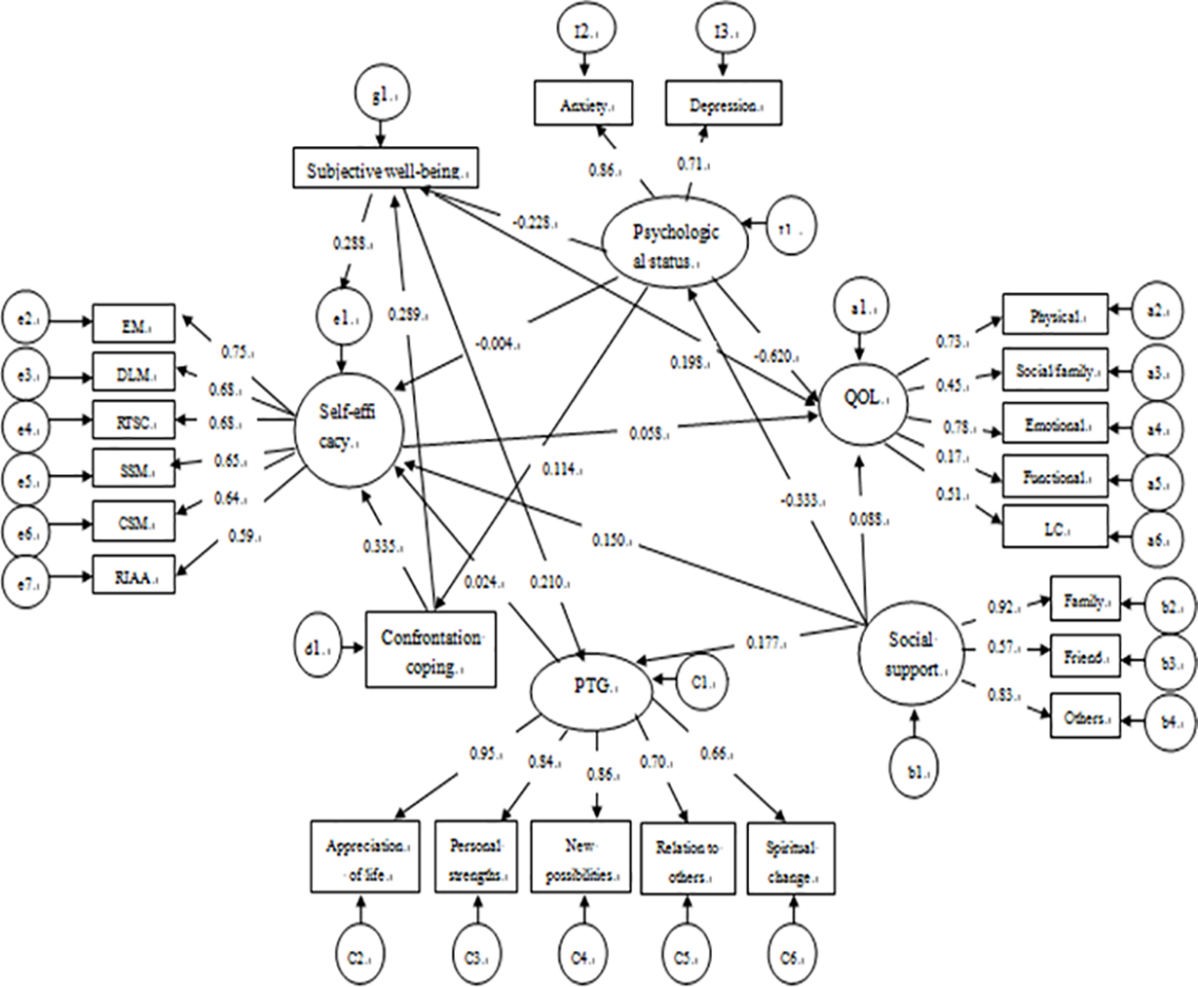
Finalized model of factors influencing self-efficacy postsurgical rehabilitation management. PTG, posttraumatic growth; LC, lung cancer subscale; EM, emotional management; DLM, daily life management; RTSC, rehabilitation training and skills cultivating; RIAA, rehabilitation information acquisition and application; CSES, coping with treatment side effects self-efficacy; SSM, symptom self-management.QOL, quality of life. Oval indicates latent variables. Square indicates observe variables. Circle indicates error. Numbers on the arrows are coefficients of influence, with the signs indicates enhancing or reducing effects.

Paths with no significant statistical effect, and variables with squared multiple correlations of 0 were eliminated from the hypothesis model (*p*>0.05).The overall fit of final model was found to be acceptable (Fig 2). The final model fit the data adequately and satisfied our preset criteria, χ2/df = 1.511(*p*>0.05), RMSEA = 0.063, CFI = 0.910, GFI = 0.900, NFI = 0.870. All paths showed significant statistical effect (*p*<0.05).

Table 2 illustrate the relationship between the structural parameters. The confrontation coping (coefficient = 0.335), SWB (coefficient = 0.288), social support (coefficient = 0.150), PTG (coefficient = 0.024) and anxiety and depression (coefficient = -0.004) can be seen to be directly related to self-efficacy (*p*<0.01 for model) (S1 Data).

**Table 2:** Effect coefficients of model-standardized estimates.

Dependent variables	Independent variables	Direct effect	Indirect effect	Total effect
Confrontation coping	Self-efficacy	0.335*	0.085*	0.419*
Social support	Self-efficacy	0.150*	0.006*	0.162*
Subjective well-being	Self-efficacy	0.288*	0.005*	0.293*
PTG ^a^	Self-efficacy	0.024*	0.000	0.024*
Anxiety and depression	Self-efficacy	-0.004*	-0.027*	-0.031*
Self-efficacy	QOL ^b^	0.058*	0.000	0.000

a, PTG, Posttraumatic growth

b, QOL, quality of life

*p<0.01

Confrontation was indirectly related to self-efficacy, mediated by SWB (indirect coefficient = 0.085, *p*<0.01). SWB was also indirectly related self-efficacy, mediated by PTG (indirect coefficient = 0.005, *p*<0.01). Social support had two indirect relationship pathways on self-efficacy, which were mediated by anxiety and depression and PTG (indirect coefficient for indirect effect = 0.006, *p*<0.01). Similar to social support, anxiety and depression also in turn had two indirect pathways to self-efficacy, via confrontation coping and SWB (indirect coefficient = -0.027, *p*<0.01).

## Discussion

This study has two unique contributions. First, we provided a comprehensive model that illustrates the relationships between diverse variables and self-efficacy for postsurgical rehabilitation management in NSCLC patients. Second, we examined the potential effect mechanisms and interactions among these factors by SEM. We found that confrontation, social support, and PTG predicted higher levels of self-efficacy, whereas anxiety and depression predicted lower levels. Therefore, the study opens new doors of enhancing self-efficacy among postsurgical NSCLC patients that emphasizes the role of confrontation coping styles, social support, SWB, PTG and anxiety and depression. We also illustrated the close relationship between self-efficacy and QOL.

Compared with avoidance and acceptance-resignation coping strategies, confrontation coping facilitated self-efficacy, and had the largest effect coefficient. Our model suggest that confrontation not only directly enhances postoperative rehabilitation self-efficacy, but also exerts indirect effects through improving the patients’ SWB. It is worthwhile to assess NSCLC populations' coping strategies as rehabilitation progresses. Healthcare providers can encourage NSCLC patients to take on corrective confrontation coping strategy in order to have a better sense of well-being and feel self efficient.

SWB was both a direct influencer and mediating factor for self-efficacy. We observed that in postsurgical NSCLC patients, SWB may further improve their cognitive function (e.g., susceptibility, comprehension, and acceptability), cognitive and problem solving initiatives, effectively strengthening the postsurgical rehabilitation self-efficacy. Some of these cognitive changes were important contents of PTG [37].

Consistent with Bandura's social cognitive theory, we noted social support as a direct facilitator of self-efficacy and indirectly enhances it through psychological states and PTG. Social support provides a feeling of psychological wellbeing, positive perceptions and growth in cancer patients [38]. Social support was viewed as a protective factor against negative psychological states (e.g., anxiety and depression) and PTG [39]. Thus, postsurgical NSCLC populations with higher level of perceived social support and PTG, and less anxiety and depression, may feel more confident to conduct postsurgical rehabilitation management activities.

Social support and self-efficacy as important facilitators of PTG have been mentioned previously [40,41], and confirmed here. We also show an indirect pathway between social support and self-efficacy via PTG. The help and support from spouse, family, and friends are a critical part of lung cancer patients' response to their illness and treatments and should be exploited to enhance self-efficacy during post-surgical rehabilitation. Construction of a support network for the NSCLC population, a platform for communication with each other, experience sharing and family encouragement may help reduce cancer patients’ postoperative anxiety and depression, and provide them a sense of hope and confidence, resulting in a greater sense of personal strength and growth.

Our study verified the direct relationship between PTG and self-efficacy, as reported previously [42]. PTG may help assist the development of self-efficacy. Postsurgical NSCLC patients with higher level of PTG tend to use social resources and personal abilities (e.g., rehabilitation coping techniques), and adapt positive coping strategies with various of physical and psychological distress.

Negative psychological states such as anxiety or depression both directly and indirectly reduced postsurgical NSCLC patients’ self-efficacy. Cancer patients with lower level of anxiety and depression tend to take positive confrontation coping strategies [43,44], and have higher SWB [45]. Healthcare providers can help postsurgical NSCLC patients increase positive emotional experiences, reduce anxiety and depression, increase their SWB and encourage them to apply more confrontation coping strategies, in order to enhance their self-efficacy.

This study is not free from limitations. First, given the cross-sectional and self-reported study design, we cannot infer causality among the factors examined. Second, the current sample was recruited from five tertiary hospitals in Fujian province and may have limited generalizability. Third, the participants have to answer roughly 150 questions in ten questionnaires that were used in this study, possibly causing survey fatigue. Finally, although the sample size met with the criterion of SEM, the same data was used to identifying the hypothesized model and evaluating the model. Future study should be designed to replicate the current study using a large sample drawn from different regional hospitals to further verify our results.

## Conclusions

This study model suggested that higher confrontation coping style, SWB, social support, and PTG and lower anxiety and depression levels could effectively enhance their self-efficacy and consequently, improve QOL. The findings could inspire the development of an effective and operative intervention aimed at enhancing self-efficacy of postsurgical NSCLC patients. To achieve this, clinical nurses should consider the following factors, such as coping skills, SWB, social support, PTG and anxiety and depression to enhance postsurgical rehabilitation self-efficacy and ultimately improve QOL.

## Supporting information

S1 Fig Approval of ethical review (Chinese version). (JPG)

S1 File Approval of ethical review (English version). (DOCX)

S1 Data Model data. (SAV)
