# Supplementary figures and images for: The structural equation model on self-efficacy during post-op rehabilitation among non-small cell lung cancer patients
Source: PLoS One. 2018 Sep 20;13(9):e0204213. doi: 10.1371/journal.pone.0204213 (PMC6147632; doi:10.1371/journal.pone.0204213)

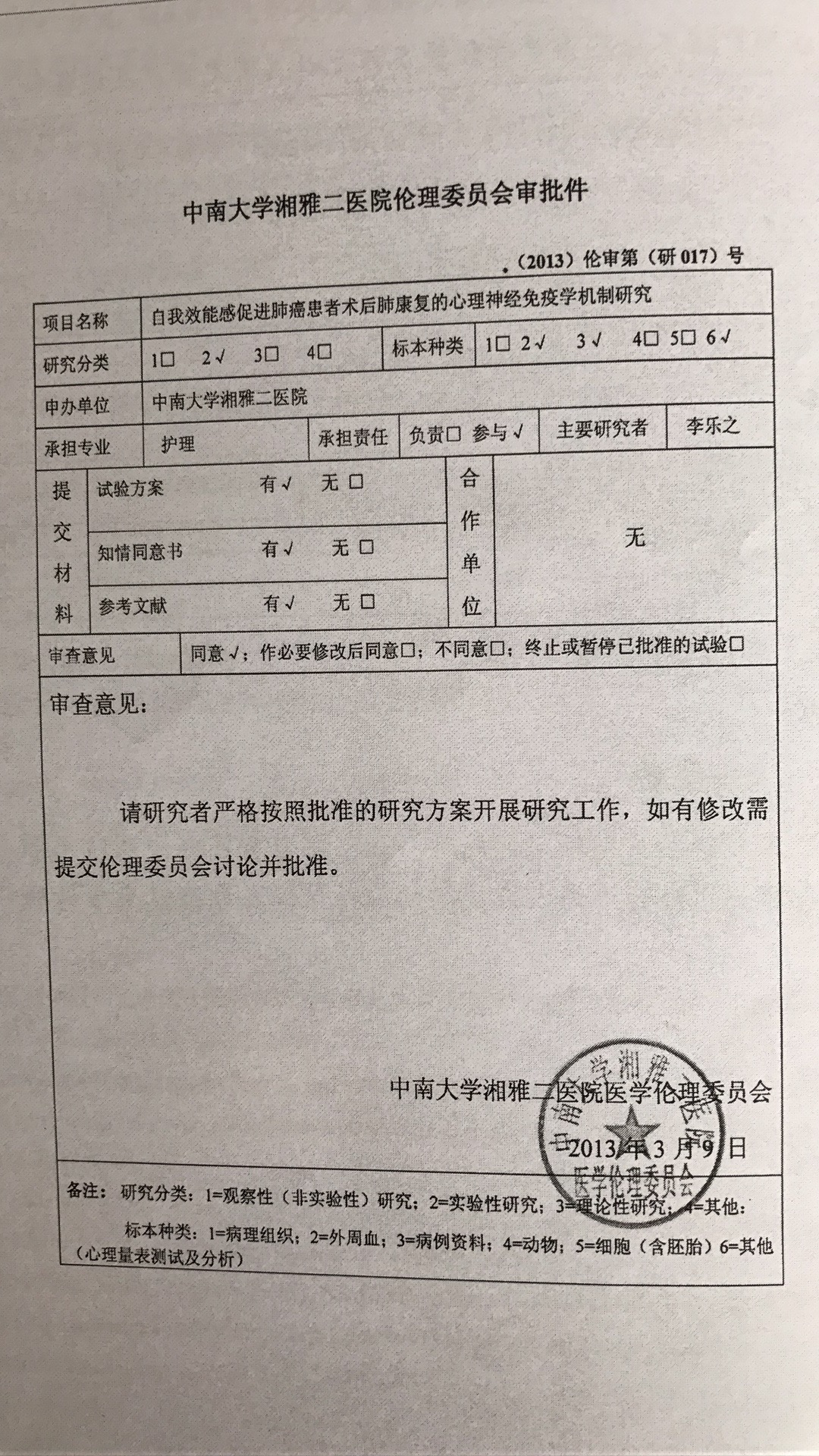

Supplement: S1 Fig — (JPG) [file pone.0204213.s001.jpg]
