# Supplementary material for: The structural equation model on self-efficacy during post-op rehabilitation among non-small cell lung cancer patients
Source: PLoS One. 2018 Sep 20;13(9):e0204213. doi: 10.1371/journal.pone.0204213 (PMC6147632; doi:10.1371/journal.pone.0204213)
Supplement: S1 File — (DOCX) [file pone.0204213.s002.docx]

The Second Xiangya Hospital of Central south university

To Whom It May Concern:

The research project entitle" The [psychoneuroimmunological](javascript:void(0);) mechanism of self-efficacy during post-op rehabilitation among non-small cell lung cancer patients" was reviewed and approved by the independent ethics committee of the Second Xiangya Hospital of Central South University in March, 9, 2013. This is to certificate that the research design and methods are in accordance with the requirements of regulations and procedures regarding to human subject protection laws as GCP and ICH-GCP.

Sincerely yours,

The Institutional Review Board

The Second Xiangya Hospital of Central south university

Date of review and approval : March 9, 2013
